# Making of voluntary evacuation maps from various disasters on hazard map 

**Published:** 2019-08

**Authors:** Toshiya Yamamoto, Kenichi Homma, Tamaho Moriwaki

**Affiliations:** ^*a*^Meiji University, Japan.; ^*b*^Odawara Junior College, Japan.

## Abstract

**Background:**

In recent years, local governments in Japan have prepared various hazard maps and distributed ‎them to residents in preparation for earthquakes and heavy rainfall. However residents are taken ‎as other persons, and the residents tend to not understand the dangers of disasters and the ‎necessity of evacuation. Therefore, JCI（Junior Chamber International）Japan started ‎activities to spread the method of making voluntary evacuation maps called Nige-Chizu map ‎throughout Japan. The purpose of making “Nige Chizu” map is to properly recognize the ‎disaster risk in the area and to promote risk communication regarding evacuation methods. It ‎can be applied not only to evacuation from tsunamis, but also to floods and landslides. ‎Therefore, we will clarify the results obtained in the workshop for JCI Japan members. ‎

**Methods:**

We gathered members of JCI around Kyoto in February 2019 and made “Nige Chizu” map of ‎walking and caring from the catastrophic flood in Fukuchiyama, Kyoto. “Nige Chizu” map ‎visualizes the time required to reach the evacuation site color-coded every three minutes based ‎on the hazard map. We made 10 types of “Nige Chizu” map with different evacuation ‎conditions, and took a questionnaire for the participants. ‎

**Results:**

According to the results of the questionnaire, although it was “Nige Chizu” map of the town ‎which many participants did not know, they answered that it was very useful in recognizing ‎disaster safety as their own problem. Through making “Nige Chizu” maps, the participants ‎considered the need for evacuation buildings from floods, consideration particularly for people ‎vulnerable to disasters, and the importance of discussions on evacuation. Then, many ‎participants answered that they would like to make “Nige Chizu” map in their own area next ‎time. ‎

**Conclusions:**

Through this workshop, it became clear that making a relief map was effective in understanding ‎the hazard map as their problem. In addition, it was revealed that the network organization of ‎leading generations like JCI is useful for spreading social technology of making “Nige Chizu” ‎map. It is expected that JCI members who have acquired social technology for making relief ‎maps will be active in Safe Community activities in various places.‎

**Keywords:**

Risk Communication, Hazard Map, Voluntary Evacuation Behavior, Workshop, Social ‎Technology, Youth Network ‎

